# Dual-Band Antenna Array Fed by Ridge Gap Waveguide with Dual-Periodic Interdigital-Pin Bed of Nails

**DOI:** 10.3390/s24165117

**Published:** 2024-08-07

**Authors:** Boju Chen, Xiaoming Chen, Xin Cheng, Yiran Da, Xiaobo Liu, Steven Gao, Ahmed A. Kishk

**Affiliations:** 1 School of Information and Communications Engineering, Xi’an Jiaotong University, Xi’an 710049, China; leochan11@stu.xjtu.edu.cn (B.C.); chengx@stu.xjtu.edu.cn (X.C.); dyr916@stu.xjtu.edu.cn (Y.D.); liu10052118@163.com (X.L.); 2 Department of Electronic Engineering, The Chinese University of Hong Kong, Hong Kong 999077, China; scgao@ee.cuhk.edu.hk; 3 Department of Electrical and Computer Engineering, Concordia University, Montreal, QC H3G 1M8, Canada; ahmed.kishk@concordia.ca

**Keywords:** antenna array, dual-band antenna, gap waveguide (GW), ridge gap waveguide (RGW), K-band, Ka-band

## Abstract

A dual-band (K-/Ka-band) antenna array is presented. An ultra-wideband antenna element in the shape of a double-ridged waveguide is used as a radiation slot, and a novel dual-periodic ridge gap waveguide (RGW) with an interdigital-pin bed of nails (serving as a filter) is used to realize dual-band operation. By periodically arranging the pins of two different heights in two dimensions, the proposed RGW with interdigital-pin bed of nails is able to realize and flexibly adjust two passbands. The widely used GW-based back cavity boosts the realized gain and simplifies the feed network design. A 4 × 4 prototype array was designed, fabricated, and measured. The results show that the array has two operating bands at 24.5–26.4 GHz and 30.3–31.5 GHz, and the realized gain can reach 19.2 dBi and 20.4 dBi, respectively. Meanwhile, there is a very significant gain attenuation at stopband.

## 1. Introduction

With the rapid development of wireless communication technology, more and more spectrum resources are used, which requires some antennas to work in multiple bands. Dual-band antennas are typically designed as shared-aperture structures to meet the market demand for small size, lightweight, and low cost [1]. A straightforward method for the shared-aperture antenna is to place antennas of different bands in the same aperture [2,3,4], which faces problems such as mutual coupling and radiation pattern distortion. Considering that multiple modes can generate corresponding resonance points at different frequencies [5], another design method for shared-aperture antennas is based on the principle of multi-resonance [6,7,8,9]. However, the antennas designed by this method generally use the printed circuit board (PCB) process, which cannot avoid the dielectric loss caused by the dielectric substrate, and this problem will be more obvious in the millimeter-wave band.

The gap waveguide (GW) proposed by P-S. Kildal et al. [10] brought a new solution for millimeter-wave device design. GW is a kind of full-metal structure based on the electromagnetic band gap (EBG) principle, which has the advantages of high power capacity, low loss, and does not require electrical contact between metal layers, so it has been applied to the design of millimeter-wave antenna arrays in a large number of applications [11,12,13,14,15,16]. A full-metal dual-band array based on GW technology was proposed in [17], which was excited by two stacked corporate-feeding networks operating in two bands. Vosoogh et al. [18] presented an antenna array with dual-band operation using a GW-based diplexer. However, for the former, two layers of GW need to be designed separately, which increases the design complexity. For the latter, introducing a diplexer results in increased insertion loss and decreased gain.

Here, a new type of ridge GW (RGW) with a dual-period interdigital-pin bed of nails (serving as a filter) is introduced to realize a dual-band antenna design. Specifically, the dual-band radiation is realized by combining a corporate-feeding network with pass-stop-pass characteristics and an ultra-wideband antenna. A one-layer corporate-feeding network is used with a back cavity, simplifying the design and improving the realized gain. This is the first time that the interdigital-pin bed of nails has been applied to antenna array design, and its dual-periodicity has been utilized to achieve filtering characteristics, which also provide a low profile, lightweight, and low processing complexity. A 4 × 4 array is designed, fabricated, and measured to verify the design concept.

## 2. Dual-Periodic Interdigital-Pin Ridge Gap Waveguide

**Figure 1:**
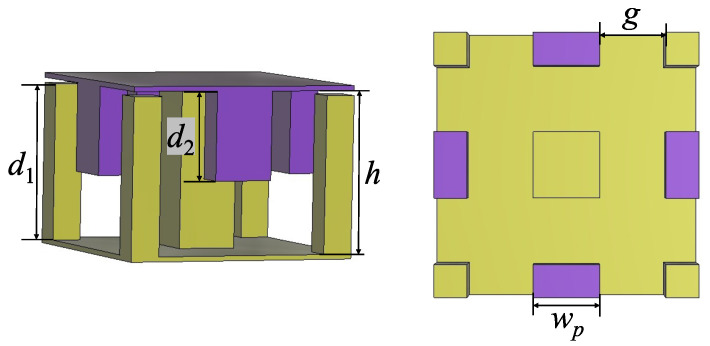
Dual-periodic interdigital-pin GW unit cell with $d_{1}=3.2\;\mathrm{mm}$, $d_{2}=1.8\;\mathrm{mm}$, $w_{r}=1.6\;\mathrm{mm}$, $w_{p}=g=1.2\;\mathrm{mm}$, and h=3.3mm.

The small physical dimensions of millimeter-wave devices make them more difficult to process, especially for complex structures like gap waveguides. GW with an interdigital-pin bed of nails presented in [19] doubles the diameter of the milling cutter, providing a high tolerance for assembly errors. Thus, it is possible to adjust the pin heights on one of the layers to achieve richer dispersion characteristics, in which pins with two different heights are staggered as one-dimensional rows [20]. Here, different pins are arranged in two dimensions, i.e., staggered in both the x- and y-directions. This new type of GW is called dual-periodic interdigital-pin GW due to the presence of two pin heights and arranged periodically. Figure 1 presents the geometry of the dual-periodic interdigital-pin GW unit cell, where different colors are used to indicate the two metal layers.

**Figure 2:**
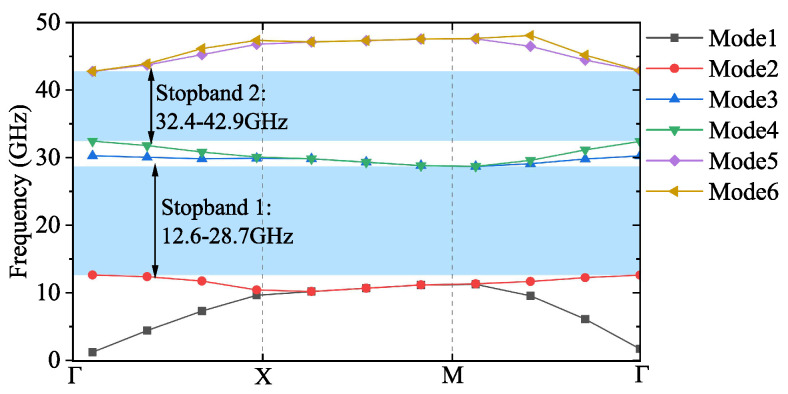
Dispersion diagram of the unit cell given by Figure 1.

**Figure 3:**
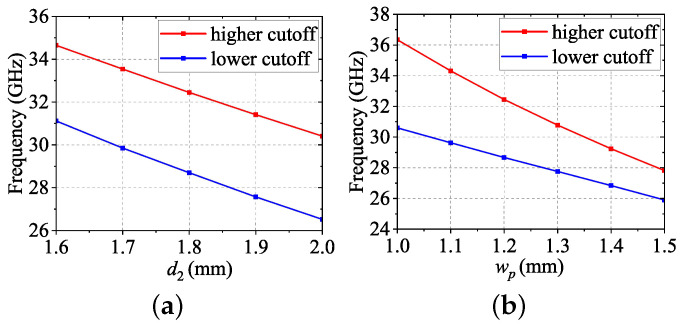
(**a**) Effect of $d_{2}$ on the cutoff frequency of Mode 3 and Mode 4 with $w_{p}=g=1.2$ mm. (**b**) Effect of $w_{p}$ on stop band with $d_{2}=1.8\;\mathrm{mm}$.

Figure 2 shows the dispersion diagram of the cell in Figure 1 calculated by CST MWS. Two stopbands appear in 12.6–28.7 GHz and 32.4–42.9 GHz, respectively. When $d_{1}$ is fixed, the position and bandwidth between the two stopbands can be adjusted by changing $d_{2}$ and $w_{p}$. Figure 3a,b show the variation of the cutoff frequencies of Mode 3 and Mode 4, respectively, when other parameters are consistent with that in Figure 1 except for $d_{2}$ and $w_{p}$. Specifically, the cutoff frequencies will shift toward lower frequencies as $d_{2}$ increases, while the bandwidth drops from 17% to 7% as $w_{p}$ varies from 1.0 to 1.5 mm.

**Figure 4:**
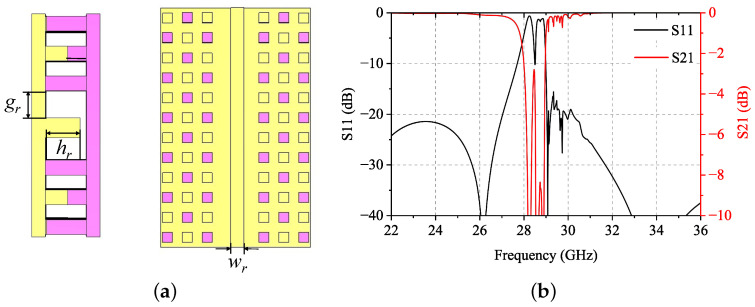
Dual-periodic interdigital-pin RGW with $w_{r}=1.6\;\mathrm{mm}$, $h_{r}=2.8\;\mathrm{mm}$, and $g_{r}=2\;\mathrm{mm}$: (**a**) geometry and (**b**) simulated S-parameters.

Figure 4a presents the geometry of RGW of height $h_{r}$ = 2.8 mm surrounded from both sides by three rows of dual-periodic interdigital-pin cells shown in Figure 1. Figure 4b presents the simulated S-parameters. Due to the dual-periodic interdigital-pin bed of nails acting as an EBG structure, the presented RGW is similar to a band-stop filter. It has a significant stopband at about 27–30 GHz, whereas it is able to achieve good electromagnetic transmission characteristics in lower and higher bands. It can be observed that the stopband of the RGW is shifted downward by about 2 GHz compared to the EBG unit cell dispersion diagram shown in Figure 2. This is because the boundary conditions around the unit cell are simulated as a periodic boundary. This ideal periodic arrangement is disrupted by the ridge of the RGW so that the cutoff frequency of each mode is shifted a little.

## 3. 2 × 2-Element Subarray Design

**Figure 5:**
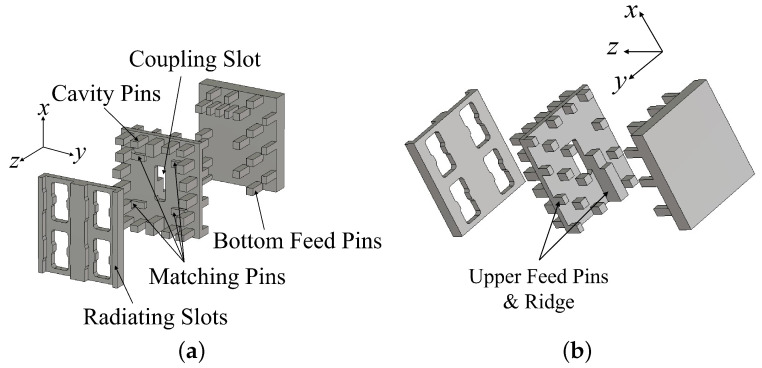
(**a**) Front view and (**b**) back view of the exploded proposed 2 × 2-element subarray.

The unit cell of the proposed array is a 2 × 2-element subarray, which consists of three metal layers, as shown in Figure 5. The feed line is based on a dual-period interdigital-pin RGW, which has half of the pins on the bottom layer, while the other half of the pins and the ridge on the back of the middle layer. The front of the middle layer is the GW-based cavity, and 2 × 2 double-ridged radiating slots are on the top layer and a rectangular coupling slot placed at the center of the middle layer.

**Figure 6:**
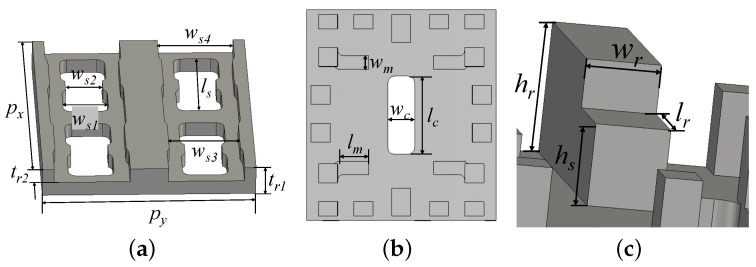
Geometries of the antenna parts: (**a**) radiating slots on the top layer; (**b**) top view of the cavity. (**c**) End of the ridge of the feed line ($p_{x}=15.6\;\mathrm{mm}$, $p_{y}=14\;\mathrm{mm}$, $w_{s1}=3\;\mathrm{mm}$, $w_{s2}=2.47\;\mathrm{mm}$, $w_{s3}=4.68\;\mathrm{mm}$, $w_{s4}=5\;\mathrm{mm}$, $l_{s}=6.3\;\mathrm{mm}$, $t_{r1}=2\;\mathrm{mm}$, $t_{r2}=1\;\mathrm{mm}$, $w_{r}=1.6\;\mathrm{mm}$, $h_{r}=2.8\;\mathrm{mm}$, $h_{s}=1.75\;\mathrm{mm}$, $l_{r}=2.3\;\mathrm{mm}$, $w_{m}=1\;\mathrm{mm}$, $l_{m}=2.3\;\mathrm{mm}$, $l_{c}=6\;\mathrm{mm}$, $w_{c}=2\;\mathrm{mm}$).

The corporate-feed network is based on RGW with an interdigital-pin bed of nails, and a one-stage stepped structure is set at the end of the ridge for impedance matching. A coupling slot is provided in the center of the middle layer to couple electromagnetic waves into the cavity. As in conventional ridge waveguides, the main transmission mode in RGW is the TE10 mode, which has a magnetic field at the coupling slot with a predominantly y-component. The design of double-ridged radiating slots is inspired by reference [14] to obtain as wide a bandwidth as possible. To widen the bandwidth by making the field distribution uniformly at lower frequencies, the cavity is provided with four matching pins, first used in reference [11], corresponding to the positions directly below each radiating slot. The above treatments are specified in Figure 6.

**Figure 7:**
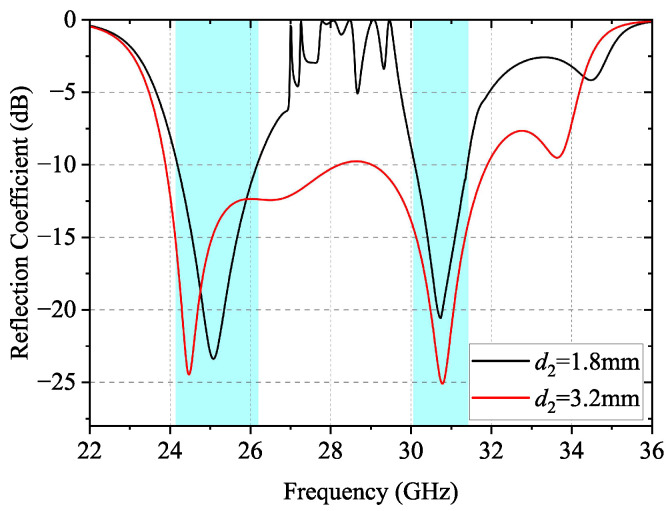
Simulated reflection coefficients of the subarray with $d_{1}=3.2\;\mathrm{mm}$ and different $d_{2}$.

The simulated reflection coefficient of the subarray with different heights of pins is shown in Figure 7. When a conventional interdigital pin is employed for the feed line, i.e., $d_{1}=d_{2}=3.2\;\mathrm{mm}$, there is no stopband in the passband of the feed line. Thus, the proposed subarray is an ultra-wideband antenna with a relative bandwidth of 28.5%. Once $d_{2}$ is reduced to 1.8 mm, the stopband appears and the reflection coefficient within the stopband is above −5 dB. Meanwhile, the subarray has a bandwidth of 8% and 5% of its operating bandwidth in the K-band and Ka-band, respectively.

**Figure 8:**
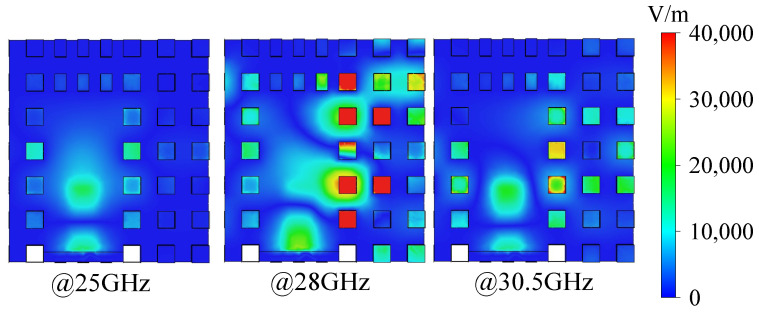
Electric field distributions at the feed line at 25, 28, and 30.5 GHz.

Figure 8 explains why a stopband exists at about 26–30 GHz. At 25 GHz, the electric field is essentially concentrated at the feed line completely, as well as at 30.5 GHz; while at 28 GHz, the electromagnetic wave is not transmitted along the ridge, but a large amount is reflected, and some of the energy also enters into the EBG structure. Even though 25 GHz and 30.5 GHz are affected by evanescent modes due to their proximity to the stopband (i.e., a small amount of electric field distribution in the EBG structure), the transmission modes are still clearly dominant in these frequencies.

## 4. 4 × 4 Array Design and Measurement

**Figure 9:**
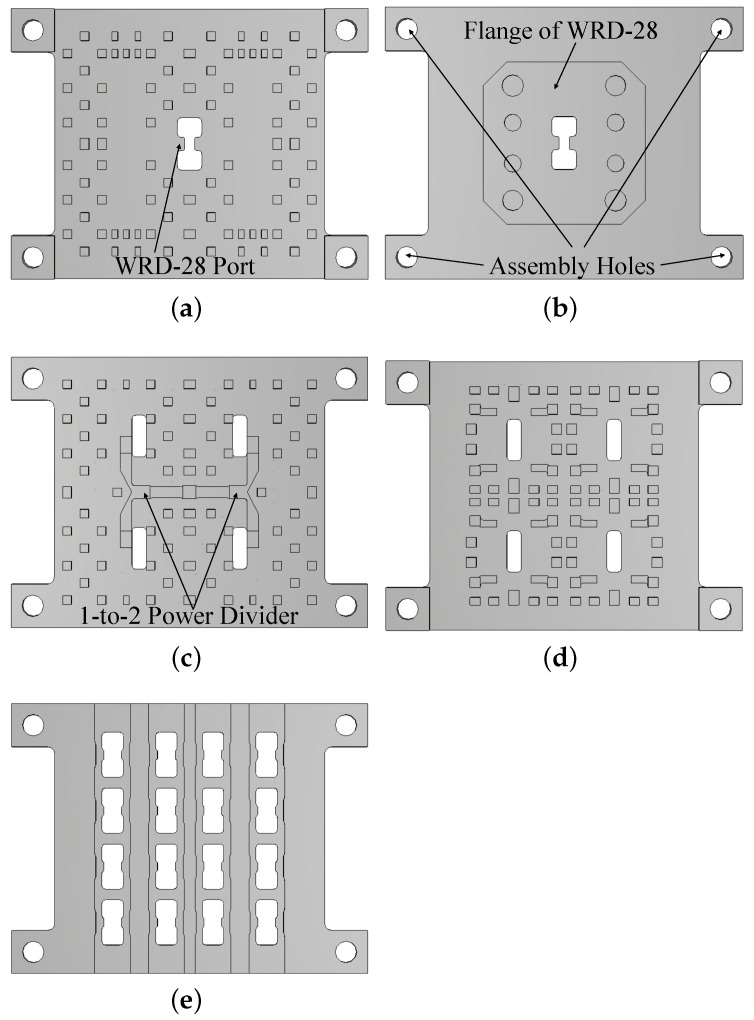
Configuration of the proposed array: (**a**) back view and (**b**) front view of the bottom layer; (**c**) back view and (**d**) front view of the middle layer; (**e**) the top layer.

To verify the feasibility of the proposed dual-band array, a 4 × 4 array is designed, fabricated, and measured. Figure 9 illustrates the configuration of the proposed 4 × 4 array. The array is fed by a corporate-feed network based on the dual-period interdigital-pin RGW. Some of the metal pins are slightly resized to fit the overall structure of the full array. In order to achieve equal amplitude and in-phase feeds for all elements, the feed network consists of two symmetrical 1-to-2 equal power dividers with some optimization of the width and height of the ridge for impedance matching. The only input port to the feed network is a centrally located WRD-28 double-ridge waveguide port on the bottom layer, which operates across the entire K- and Ka-band. Placing the input port on a separate layer from the feed line conserves space within the middle layer, thus ensuring that the array can be further expanded to 8 × 8 or larger. A standard flange of WRD-28 is provided on the back of the bottom layer for connection to the feeding waveguide.

**Figure 10:**
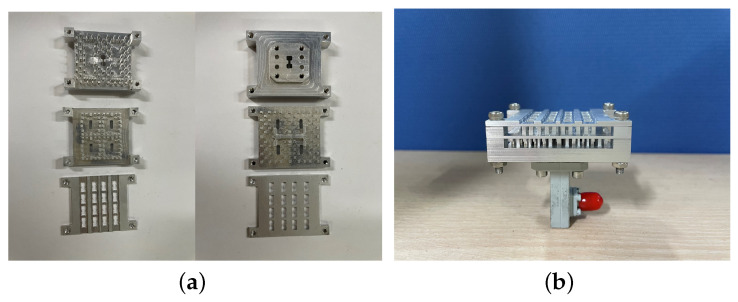
Photograph of the fabricated prototype: (**a**) front view (left) and back view (right) of each layer; (**b**) side view of the prototype with a waveguide-to-coaxial converter.

Finally, the whole array was fabricated by CNC milling technology using only aluminum (shown in Figure 10). The dimensions of the prototype are 37.6 × 49.6 × 14.6 ${\mathrm{mm}}^{3}$. In the reported works [21,22,23], it has been well demonstrated that microwave and millimeter-wave components based on gap waveguide technology require only some screws for assembly. In addition, according to the misalignment analysis in reference [19], the interdigital-pin GW is highly tolerant to assembly errors in the x- and y- directions. Therefore, the prototype array is equipped with four assembly holes only at the corners, and the three metal layers are fixed together by screws. The proposed array was also simulated in CST MWS.

**Figure 11:**
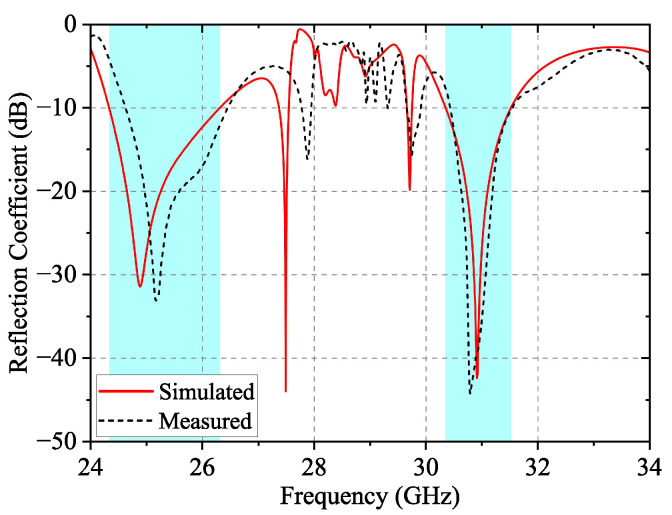
Simulated and measured reflection coefficients of the prototype array.

The simulated and measured reflection coefficient of the 4 × 4 array is presented in Figure 11, which is in great agreement. At 24.5–26.4 GHz in the K-band and 30.3–31.5 GHz in the Ka-band, the measured reflection coefficient is less than −10 dB, and the relative impedance bandwidth is 7.47% and 3.88%, respectively.

After forming the full 4 × 4 array, the reflection coefficient in the stopband fluctuates a bit. It is close to or below −10 dB because part of the electromagnetic wave leaks through the surrounding EBG, but it does not form effective radiation. Therefore, it does not have an impact on dual-band performance.

**Figure 12:**
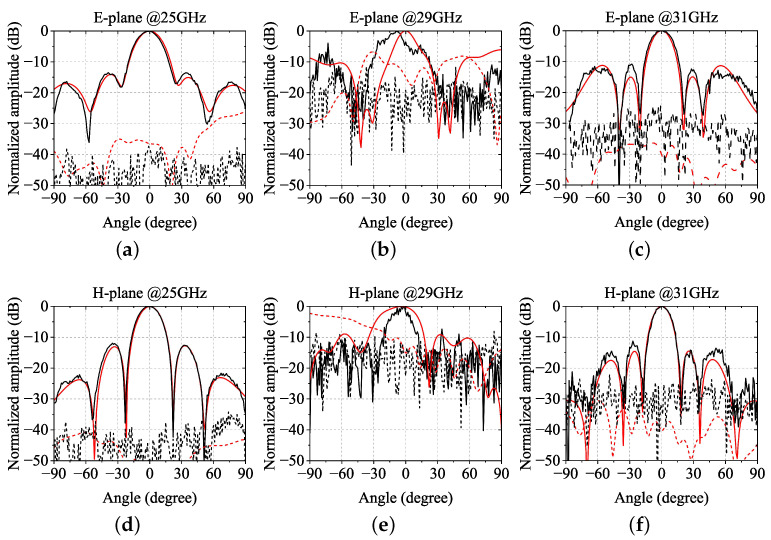
Simulated and measured radiation patterns at (**a**) 25 GHz in the E-plane, (**b**) 29 GHz (stopband) in the E-plane, (**c**) 31 GHz in the E-plane, (**d**) 25 GHz in the H-plane, (**e**) 29 GHz (stopband) in the H-plane and (**f**) 31 GHz in the H-plane. Solid lines represent co-polarization, dashed lines represent cross-polarization, red lines represent simulated results, and black lines represent measured results.

Figure 12 shows the simulated and measured results of the radiation patterns at K-band (25 GHz) and Ka-band (31 GHz) in the E-plane and H-plane. The measured results are very close to the results obtained by simulation. The cross-polarization level can be as low as about −40 dB in the K-band but rises to about −30 dB in the Ka-band. In Ka-band, the pattern shows a grating lobe in the E-plane, but it is not higher than the first side lobe in the measured results. Furthermore, in the Ka-band, the measured results show a certain amount of burr, which mainly comes from the effect of the environment of measurement. Also, simulated and measured radiation patterns in the stopband (29 GHz) are illustrated to demonstrate the filtering characteristics of the proposed array. As expected, in the stopband, the power is distributed all over the pins region, making the available power over the ridge very small, reducing the power coupled to the slots. Thus, the stopband pattern is dramatically distorted, and the cross-polarization deterioration is severe. The simulated and measured patterns differ significantly due to the noise amplification of normalization of the weak pattern.

**Figure 13:**
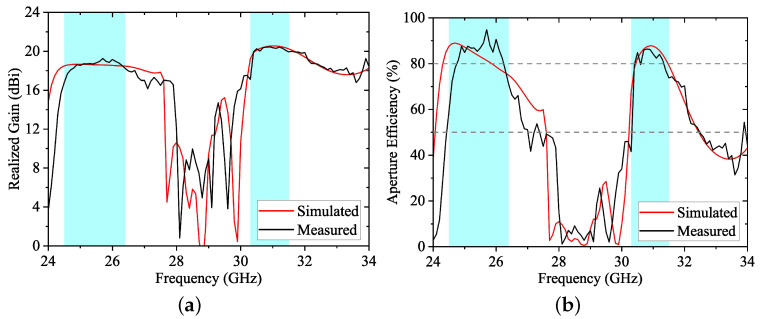
(**a**) Simulated and measured realized gains of the prototype array. (**b**) Simulated and measured aperture efficiencies of the prototype array.

Figure 13 illustrates the broadside realized gains and aperture efficiencies obtained from simulation and measurement. The aperture efficiency here is calculated by the following equation:

$$\begin{array}{c} \eta_{ape}=\frac{G\lambda^{2}}{4\pi S}, \end{array}$$

where $\eta_{ape}$ is aperture efficiency, *G* is the broadside gain, λ is the free-space wavelength, and *S* is the array size. It can be seen that the proposed array can achieve aperture efficiency higher than 80% in both operating bands. Particularly, the attenuation of gain is significant outside the operating bands. The aperture efficiency of the prototype is well below 50% within the expected stopband (within about 28–30 GHz), demonstrating excellent band-stop performance.

The simulated and measured gains, radiation efficiencies, and aperture efficiencies at several frequencies are shown in Table 1. Table 1 reveals a measured peak gain of 19.16 dBi in the lower band and 20.37 dBi in the higher band, with radiation efficiency over 90% and aperture efficiency above 84% in both operating bands. Meanwhile, there are significant attenuations of efficiencies as well as of gains in the stopband.

Table 2 demonstrates various performance comparisons of the proposed array with other reported dual-band antenna arrays based on GW. It can be seen that the proposed antenna has a very significant advantage in terms of gain and aperture efficiency.

**Figure 14:**
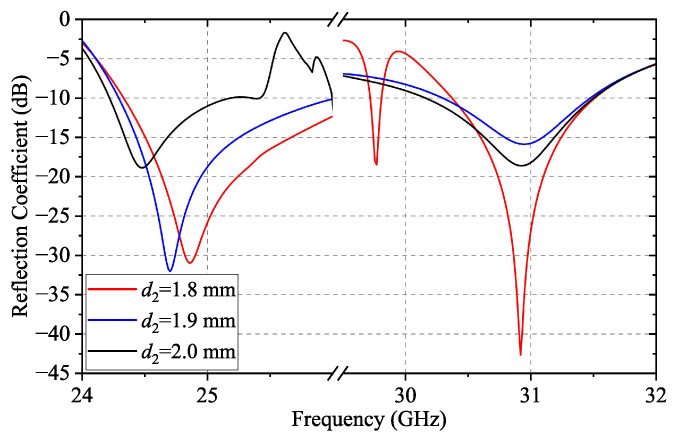
Simulated reflection coefficient of the proposed array at different $d_{2}$ when $w_{p}=1.2\;\mathrm{mm}$.

The fact that the stopband of the dual-periodic RGW can be tuned by changing the key dimensions suggests that the two operating bands of the proposed dual-band array can be flexibly tuned in the same way. Figure 14 demonstrates the simulated reflection coefficient of the proposed array at different $d_{2}$ when $w_{p}=1.2\;\mathrm{mm}$. As $d_{2}$ increases, the stopband is shifted to the lower frequency, which reduces the bandwidth of the lower operating band while the bandwidth of the higher operating band is increased.

## 5. Conclusions

A 4 × 4 K-/Ka-band dual-band antenna array has been proposed. By feeding an ultra-wideband antenna element with a dual-periodic interdigital-pin RGW (serving as a filter), dual-band operation has been achieved using only one layer of the feed network and without a diplexer. Simulated and measured results have shown that the antenna array has performed well in both K-band and Ka-band. The proposed array has achieved 90% radiation efficiency and 84% aperture efficiency, making it a good choice for dual-band antennas in future wireless communication systems. Thanks to the configuration in which the input port and the feed network are placed on different metal layers, this technology has the potential to be extended to larger array designs.

## Figures and Tables

**Table 1:** Simulated and measured gains, radiation efficiencies, and aperture efficiencies at different frequencies.

Freq. (GHz)	Sim. Gain (dBi)	Mea. Gain (dBi)	Rad. Eff. (%)	Ape. Eff. (%)
25	18.65	18.54	99.32	84.91
26	18.54	19.16	97.98	90.58
28	10.63	12.02	67.75	15.07
29	9.42	8.99	60.13	6.99
30.5	20.13	20.27	94.95	84.83
31	20.55	20.48	98.97	86.38

**Table 2:** Comparison between proposed and reported dual-band antenna arrays based on GW.

Ref.	Element Num.	Center Fre. (GHz)	Gain (dBi or dBic)	Ape. Eff. (%)
[17]	8 × 8	20; 30	26; 29.5	83; 85
[24]	4 × 4	20; 30	16.6; 18.5	85.4; 88.6
[18]	16 × 16	28.2; 29.2	N/A	60; 60
**This Work**	**4 × 4**	**25; 31**	**19.2; 20.4**	**91; 86**

## Data Availability

All data are available from the author.
